# Comprehensive Genomic Characterization of *Campylobacter* Genus Reveals Some Underlying Mechanisms for its Genomic Diversification

**DOI:** 10.1371/journal.pone.0070241

**Published:** 2013-08-05

**Authors:** Yizhuang Zhou, Lijing Bu, Min Guo, Chengran Zhou, Yongdong Wang, Liyu Chen, Jie Liu

**Affiliations:** 1 BGI-Shenzhen, Shenzhen, Guangdong Province, China; 2 Biology Department of University of New Mexico, Albuquerque, New Mexico, United States of America; 3 Department of Biology, Sichuan University, Chengdu, Sichuan Province, China; 4 Key Discipline Laboratory for National Defense for Biotechnology in Uranium Mining and Hydrometallurgy, University of South China, Hengyang, Hunan Province, China; 5 Department of Microbiology, Xiangya School of Medicine, Central South University, Changsha, Hunan Province, China; 6 Translational Center for Stem Cell Research, Tongji Hospital, Stem Cell Research Center, Tongji University School of Medicine, Shanghai, China; University of Helsinki, Finland

## Abstract

*Campylobacter species.*are phenotypically diverse in many aspects including host habitats and pathogenicities, which demands comprehensive characterization of the entire *Campylobacter* genus to study their underlying genetic diversification. Up to now, 34 *Campylobacter* strains have been sequenced and published in public databases, providing good opportunity to systemically analyze their genomic diversities. In this study, we first conducted genomic characterization, which includes genome-wide alignments, pan-genome analysis, and phylogenetic identification, to depict the genetic diversity of *Campylobacter* genus. Afterward, we improved the tetranucleotide usage pattern-based naïve Bayesian classifier to identify the abnormal composition fragments (ACFs, fragments with significantly different tetranucleotide frequency profiles from its genomic tetranucleotide frequency profiles) including horizontal gene transfers (HGTs) to explore the mechanisms for the genetic diversity of this organism. Finally, we analyzed the HGTs transferred via bacteriophage transductions. To our knowledge, this study is the first to use single nucleotide polymorphism information to construct liable microevolution phylogeny of 21 *Campylobacter jejuni* strains. Combined with the phylogeny of all the collected *Campylobacter* species based on genome-wide core gene information, comprehensive phylogenetic inference of all 34 *Campylobacter* organisms was determined. It was found that *C. jejuni* harbors a high fraction of ACFs possibly through intraspecies recombination, whereas other *Campylobacter* members possess numerous ACFs possibly via intragenus recombination. Furthermore, some *Campylobacter* strains have undergone significant ancient viral integration during their evolution process. The improved method is a powerful tool for bacterial genomic analysis. Moreover, the findings would provide useful information for future research on *Campylobacter* genus.

## Introduction

*Campylobacter* is a genus of Gram-negative bacteria that are spiral-shaped microaerophilic and motile [1]. *Campylobacter spp.*have unipolar or bipolar flagella, and generally colonize different mucosal surfaces. The genus *Campylobacter* was first proposed by Sebald *et al*. in 1963 [2]. Since then, the number of *Campylobacter* species discovered has significantly increased. The genus comprised 17 species with validly published names, including six recognized subspecies, in 2004 [3], [4]. At present, 23 species are recorded in the National Center for Biotechnology Information (NCBI) Taxonomy Division. Members of the genus *Campylobacter* colonize diverse host habitats, from livestocks to humans [5]–[11], indicating that their genomes diversify to adapt to various host environments. *Campylobacter spp.* are significantly diverse with regard to their pathogenicity. Some species definitely cause human disease. *C.jejuni* is one of the most important food-borne pathogen in the world, and its infection is a leading cause of acute bacterial diarrhea in humans in many developed countries [12], [13]. *Campylobacter coli*, *Campylobacter upsaliensis*, and *Campylobacter lari* are also associated with human gastroenteritis [6], [7], [14]. Some species are causative agents of pericarditis/myocarditis [15], [16] and Guillain–Barre Syndrome in humans [17]–[19]. Other *Campylobacter* species are related to severe animal diseases; for instance, *Campylobacter fetus* can cause abortion in animals [20]–[22] and bovine genital campylobacteriosis [23], [24]. However, not all *Campylobacter spp.* are pathogenic, suggesting that their phenotypic diversities in terms of pathogenicity may result from genomic diversities within the *Campylobacter* genus. Thus, comprehensive characterization of their genetic diversities that contribute to their phenotypic diversities should be conducted, and the underlying mechanisms should be determined.

Since the publication of the *Campylobacter* genome for the *C. jejuni* strain 11168, 34 *Campylobacter* genomes have been sequenced. Thus, systemic analysis and comparison of the entire *Campylobacter* genus should be conducted to illustrate its genomic diversity and provide insights into its mechanisms for genomic diversity. Although several reports have analyzed a single or few genomes [25]–[33], to our knowledge, intensive and combined investigation on the genotypic diversity of all *Campylobacter* genomes has not been conducted yet. In the present study, 34 genomes of the *Campylobacter* genus were downloaded and systemically analyzed to describe their genetic diversities. After depiction, the possible mechanisms of their genetic diversity were investigated. The genome of *C. jejuni* contain high fraction of intraspecies-originated abnormal composition fragments (ACFs), whereas that of the other *Campylobacter* members have relatively high fraction of intragenus-originated ACFs. Moreover, ancient bacteriophage integration in some organisms contributed to their genomic evolution.

## Materials and Methods

### Collection of *Campylobacter* Genomic Data

Thirty-four *Campylobacter* genomes were downloaded from the NCBI website. Among these genomes, 14 were complete, while the rest were drafts (**Table S1**). Several control genomes including *Campylobacterales bacterium* GD 1 (draft, GI: 254456697); *Methanothermobacter thermautotrophicus* str. Delta H (complete, GI: 15678031), which is an archaeal genome; and *Escherichia coli* str. K12 substr. DH108 (complete, GI: 169887498) were also collected from NCBI.

### Genome-wide Alignments

NUCleotide MUMmer (NUCmer) was used to assess the genome-wide alignments according to reference [34]. To ensure that all contigs were aligned, all maximal matches were used as alignment anchors (-maxmatch). The delta encoded alignment files produced by NUCmer were filtered using the delta-filter utility, leaving only the alignments that form the longest consistent set (-q –r –l 200). A summary of all the alignments produced by NUCmer was generated using the show-coords utility. Afterward, we developed in-house Perl scripts to calculate the pairwise-aligned genome percentages for each reference.

### Gene *de novo* Prediction

Gene *de novo* prediction was conducted for some draft genomes such as *C. jejuni* CG8486, which have no protein coding gene information in NCBI. Considering the consistency of the gene prediction among all collected genomes, protein-coding genes for all listed genomes were *de novo-*predicted or re-predicted using Glimmer 3.02 with default parameters [35]. To facilitate subsequent analysis, tRNA-coding genes were re-predicted by tRNAscan-SE (version 1.23) [36], while rRNA-coding genes were re-predicted by RNAmmer (version 1.2) [37]. Only small subunit ribosomal RNA (SSU rRNA) sequences with RNAmmer score above 1700 were used for further analysis.

### SSU rRNA Tree for the *Campylobacter* Genus

Among the several SSU rRNA sequences in specific *Campylobacter* genomes having an RNAmmer score above 1700, only the first SSU rRNA sequence was selected for the phylogenetic tree analysis. The size of all selected sequences was approximately 1500 bp, except for the representative sequence from *Campylobacter curvus* 525.92, which has an intervening sequence having a length of approximately 200 bp. After trimming the intervening sequence, all pooled sequences were subjected to multiple alignments using the software pyNAST [38]. The subprogram phyml of TreeBeST (http://treesoft.sourceforge.net/treebest.shtml) was used to construct a phylogenetic tree with default parameters. Non-parametric bootstrap analysis with a thousand re-sampling was conducted to obtain the bootstrap values for all branches.

### Protein Family Construction and Pan-genome Analysis

All protein coding gene sequences were translated into protein sequences according to Codon Table 11 from NCBI. The resultant protein sequences were clustered into gene families using BLASTclust. Protein or gene sequences were automatically and systematically clustered according to the “fifty-fifty” rule based on pairwise matches. For example, two sequences were clustered in the same family if the best local alignment between these clusters covers at least 50% of the length of both sequences and contains at least 50% identities. The in-house Perl scripts were then used to analyze the gene family distribution among the strains of *Campylobacter* genus. The protein sequences of the repeat genes of an organism and the core genes of *Campylobacter* genus plus the total representative protein sequences were extracted to conduct alignments against the Cluster of Orthologous Groups (COG) database [39] using the local alignment tool BLASTP. Only the alignments with e-value ≥1e-5 and coverage ≥50% of both query and subject sequences were used for COG annotation. Functional enrichment difference between repeat genes and total genes, as well as between core genes and total genes were evaluated by chi-square test.

All protein sequences of the entire core genes were extracted from the results. When one organism had at least two genes in one gene family, only orthologous protein sequences with the best hit for homologous genes in other organisms were chosen to construct the genome-wide phylogenetic tree. After manual adjustment to search for conserved regions, all orthologous protein sequences for each core gene family, which refers to the family with at least one gene from each of the *Campylobacter* strain, were pooled for independent multiple alignments using the MUSCLE software [40]. Conserved sequences shared by all organisms were concatenated to construct a neighbor-joining phylogenetic tree using the TreeBeST software with default parameters. Bootstrap values of all branches were obtained after 1000 times of resampling. Gene families shared by four close species, namely, *C.jejuni*, *C. coli*, *C. lari,* and *C. upsaliensis,* were obtained and represented in Venn diagram.

### Microevolution Analysis of *C. jejuni*

To identify further the phylogenetic relationships between different strains of *C. jejuni*, single nucleotide polymorphisms(SNPs) were used for phylogenetic analysis. Repeat genome fragments of *C. jejuni* NCTC11168 were previously identified using a combinatory approach based on RepeatMasker and self-Blast [41]. Each of the other strains was then aligned against the reference *C. jejuni* NCTC 11168 using NUCmer [34]. The delta-encoded alignment files generated by NUCmer were filtered using the delta-filter utility, and raw SNPs were detected using the show-snps utility. The program BLAT [42] was used to map raw SNP calls against their genomes for verification, while SNPs localized in repetitive genomic fragments were filtered out. The filtered SNPs were concatenated and aligned by ClustalW 2.0 [43]. Phylogenetic relationships based on the aligned sequences were inferred using neighbor-joining methodologies implemented in TreeBeST (default parameters) with 1000 bootstrap pseudoreplicates.

### Genome Composition Analysis

Genomic composition, namely, tetranucleotide usage patterns (TUP), was analyzed for all the 34 collected *Campylobacter* genomes and the outgroup *C. bacterium* GD 1 genome. The overlapping oligonucleotides (length, 4) of the genomes were quantified as observed tetranucleotide frequencies by shifting the window at a step length of one nucleotide. The corresponding expected values were calculated according to a previous method [44]. The differences between the observed and expected values were transformed into z-values for each tetranucleotide [45]. The similarity between genomes was assessed by calculating the Pearson correlation coefficient for the 256 tetranucleotide-derived z-values. Hierarchical cluster analysis was conducted using Ward’s method.

### Identification of ACFs

To exclude the similarity between genomes of the *Campylobacter* genus, a relatively distant genome from an archaea, *M. thermautotrophicus* str. Delta H, was used as control. All genomes were first split into contigs at positions with ambiguous nucleotides (“N”). The resultant contigs were further split into fragments (S) with length of 500 bp and 250 bp overlap. ACFs were then assessed by calculating the fold as previously described [46]: Fold = P(S:G_i_)/P(S:G), where *P* is the probability calculated by the naïve Bayesian classifier (in this study, motif length is 4); *G*_i_ is training genome; *G* is the testing genome, from which sequence *S* is derived; and *S* is a fragment with length of 500 bp for investigating abnormal composition.

A cutoff for identification of the ACFs was determined through comparison with *M. thermautotrophicus*. Any fragment above the cutoff was considered as an ACF (7, logarithmically transformed). To detect ancient viral or phage element integration into the *Campylobacter* genomes, at least two consecutive ACFs were concatenated into genomic regions and subjected to comparison with virus or phage homologous sequences. In addition, the gene types of the genomic regions were also analyzed.

### Identification of Viral and Phage Homologous Protein-coding Genes

All *Campylobacter* proteins were aligned against the NR-virus database using BLASTP. The NR-virus database was in-house constructed by extracting all virus sequences from NCBI. All hits with e-value ≤1e-5 and cover at least 50% of the length of viral sequences were used, and their corresponding query sequences were considered as virus homologous sequences.

The virus database was not exclusively developed for bacterial analysis and may lack some useful information. Phage DNA in *Campylobacter* is better defined. We found that numerous sequences that were previously annotated as phage-related sequences according to published genomes were not included in the set of our virus homologous sequences. To identify the largest possible bacteriophage sequences in *Campylobacter* strains, phage homologous sequence identification was conducted. All phage-related *Campylobacter* proteins were obtained from NCBI and pooled together as phage database. A total of 285 phage-related sequences from the NCBI database were included in the phage database. All the predicted or re-predicted protein-coding sequences were then aligned against the phage database, following the same procedure as that for the detection of virus homologous proteins.

## Results and Discussion

### Genome-wide Alignments of *Campylobacter* Genomes

**Figure 1:**
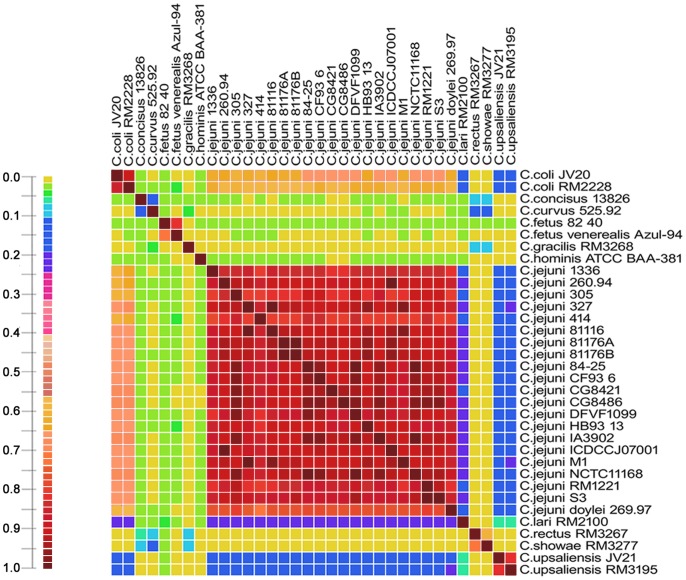
Genome-wide alignment heatmap of the genus *Campylobacter*. This figure represents the pairwise genome-wide alignment similarity of the 34 genomes in this study. The similarity ratios were calculated using the total length of the genome fragments preserved between any two organisms divided by the length of the total genomes of the organism in row. The ratio is used as a basis for color intensity. Table S1 displays the absolute ratios in detail.

To explore the diversity of *Campylobacter* genomes, we conducted genome-wide alignments for all collected *Campylobacter* genomes to provide an overview of the percentage of the conserved genomic regions between pairwise genomes. As shown in **Figure 1**, the *Campylobacter* genus exhibited genomic diversity at genus level despite the absence of conserved genomic fragments between genomes, such as *Campylobacter rectus* RM3267 and *C. coli* JV20 (for pairwise identity values see **Table S2**). Aside from the obvious genus differences, we also detected slight genomic differences between *Campylobacters* at the species level. For instance, the genomic similarity between *C. fetus* subsp. fetus 82–40 and *C. fetus* venerealis Azul-94 is 0.7 (calculated using the total length of *C. fetus* subsp. venerealis Azul-94 as the denominator) or 0.81 (calculated using the total length of *C. fetus* 82–40 as the denominator), similar to that of a previous study [32]. The diversity among the *Campylobacter* strains occurs not only at the genome level but also at the protein level, which was shown by a previous report using alignments of protein coding sequences (CDS) [47]. Therefore, some mutation events such as horizontal gene transfer (HGT) occurred after speciation from their ancestor.

### Phylogenetic Analysis Based on SSU rRNA Sequences

All draft genomes such as that of *C. jejuni* subsp. *jejuni* strains CG8486, CG8421, and DFVF1099 have SSU rRNA sequences that meet the SSU rRNA prediction criteria (**Table S1**). The length of all predicted SSU rRNA sequences are approximately 1500 bp. However, the sequence from *C. curvus* 525.92 has a length of 1701 bp because of the intervening sequences with length of approximately 200 bp, similar to that in a documented report [48]. Therefore, the intervening sequence of the *C. curvus* 525.92 representative SSU rRNA was trimmed before being pooled together with other representative SSU rRNA sequences to construct the phylogenetic tree.

**Figure 2:**
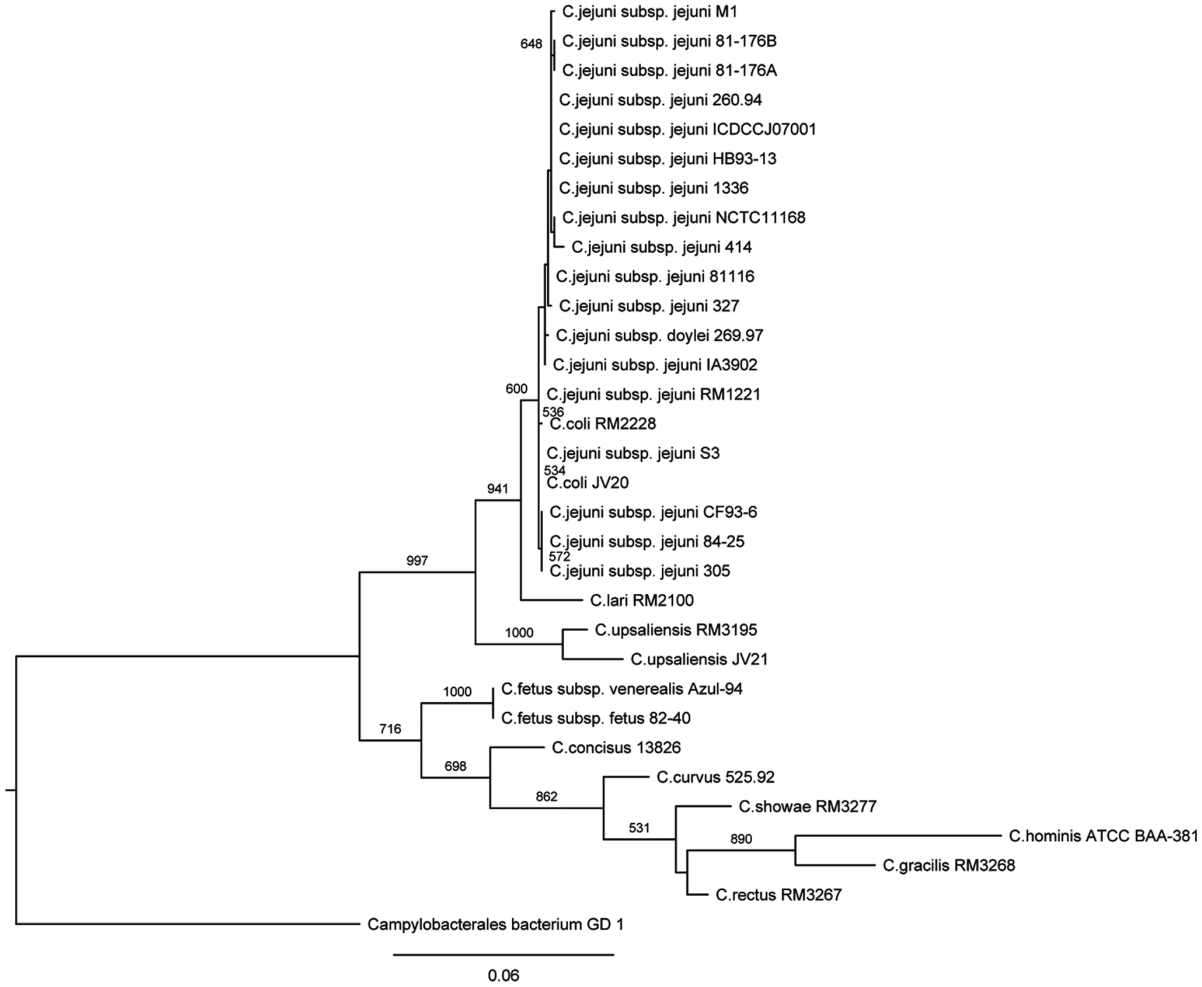
Maximum likelihood phylogenetic tree of *Campylobacters* based on small subunit ribosomal RNA (SSU rRNA) sequences. The tree is rooted with SSU rRNA sequence of *Campylobacterales bacterium* GD 1, a member of the common order of *Campylobacterales* considered as a relative close outgroup. Absolute bootstrap values of 1000 simulations are shown beside the major branches to indicate the stability of the branching. Only bootstrap values greater than 500 are shown. The scale bar represents the 0.06 nucleic acid substitutions per site.

The phylogeny based on SSU rRNA (rRNA tree) shows that *C. coli* is hardly distinguishable from *C. jejuni*, confirming that they are phenotypically and genotypically similar (**Figure 2**). This result is consistent with that by a previous study, in which the phylogeny was inferred from rpoB or SSU rRNA sequences [47]. However, C. *jejuni* is clearly different from *C. coli* in the *groEL*-based tree [49]. Furthermore, the *Campylobacter* genus can split into two main lineages, i.e., the *jejuni* lineage, which includes *C. jejuni*, *C. coli, C. lari*, and *C. upsaliensis*; and the *non-jejuni* lineage, which includes *C. fetus*, *Campylobacter concisus*, *Campylobacter gracilis*, *Campylobacter hominis*, *C. curvus*, *C. rectus*, and *Campylobacter showae*. Despite the discrepancies among different phylogenetic trees (rRNA tree, core gene tree in this study, and previously documented trees [26], [47], [49]), C. *jejuni*, *C. coli, C. lari*, and *C. upsaliensis* are always in a common cluster using different sequences or methods, suggesting that these four species are genetically close and the global topology of the *jejuni* lineage is always essentially identical, whereas that of the *non-jejuni* lineage is more or less diverse. In addition, the *jejuni* lineage is not limited to these four species; some other species, such as *Campylobacter insulaenigrae*, *Campylobacter helveticus* [47], and *Campylobacter hyoilei* [49], may also be included in this lineage.

### Pan-genome Analysis

Protein-coding genes were predicted using GLIMMER 3.02 [35]. Collectively, 64686 genes were predicted for all the 34 collected genomes. Among these genes, C. *fetus* subsp. venerealis Azul-94 harbors the most number of protein-coding genes with 2447, whereas *C. lari* RM2100 harbors the fewest protein-coding genes with 1545 (**Table S3)**. A total of 13167 gene families were obtained after clustering using BLASTclust according to the “fifty-fifty” rule mentioned earlier. Among the 13167 gene families, 348 (2.64%) are core gene families at the genus level, while the other 12819 (97.36%) are dispensable or unique gene families. Core gene families comprise 12181 protein coding genes, accounting for 18.83% of the total genes. By contrast, the dispensable or unique gene families comprise 52505 genes, accounting for approximately 81.17% of the total genes.

**Figure 3:**
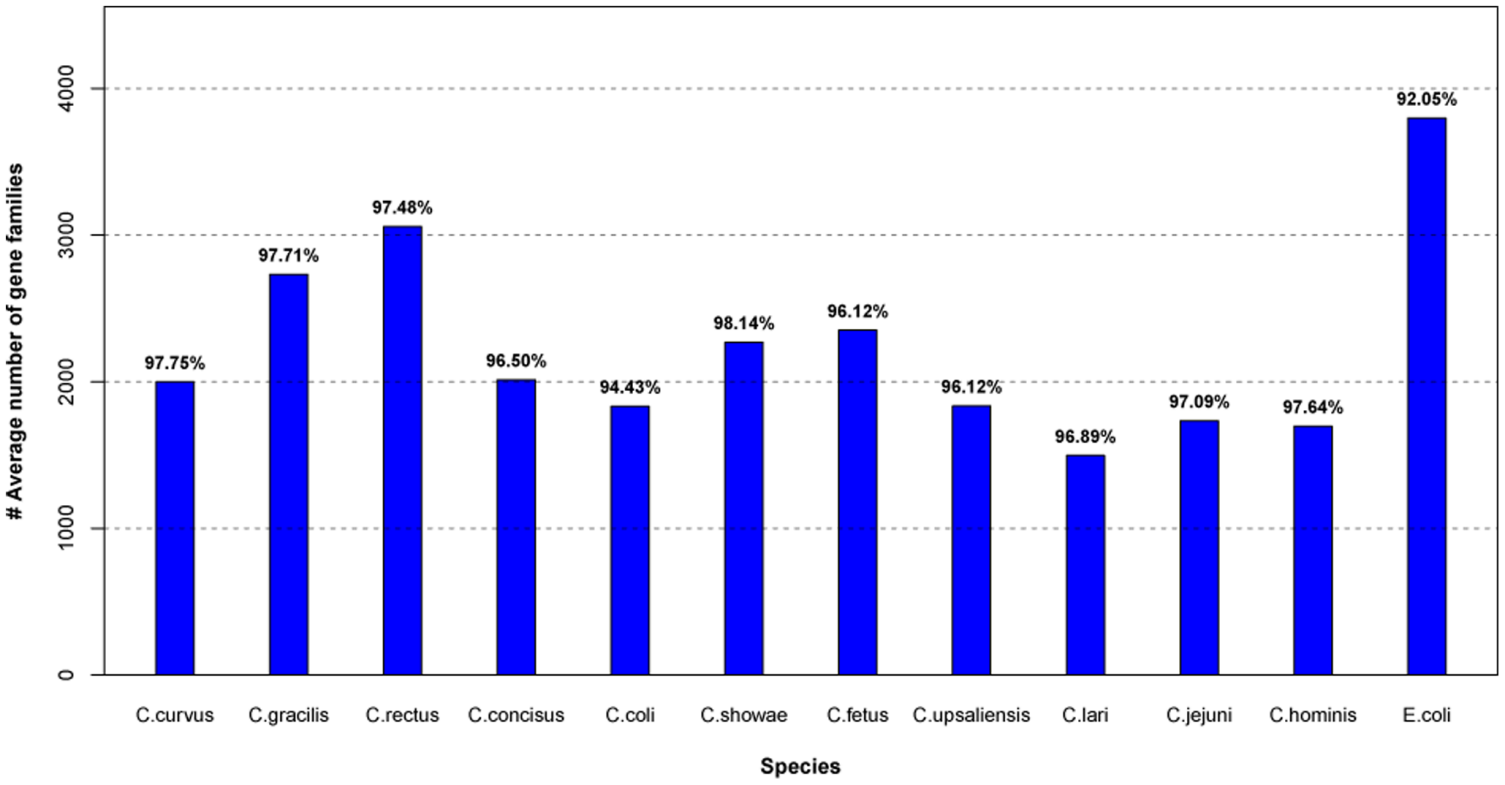
Gene families of genus *Campylobacter*. *Escherichia coli* (*E. coli*) str. K12 substr. DH108 (GenBank accession CP000948) was used as a control. The values above each bar (in percent) were calculated as the average number of gene families divided by the average number of total genes.

**Figure 4:**
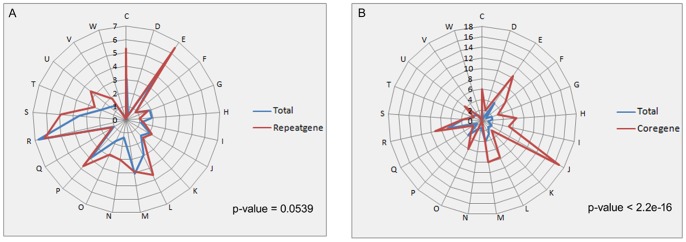
Spider charts of COG enrichment. Chi-square tests were applied to test their differences. The blue line shows the COG enrichment of the total genes, whereas the red lines indicate that of repeat genes (A) and core genes (B). A. COG enrichment of the repeat genes compared with that of the total genes. Repeat genes have no significant differences compared with the total genes (*p* = 0.0539). B. COG enrichment of core genes compared with that of total genes. COG enrichment of core genes and that of total genes (*p*<2.2e-16) was significantly different between COG enrichment.

The protein families of each *Campylobacter* species are shown in **Figure 3**. Genes from the same organism with more than one gene in a gene family were defined as repeat genes. All *Campylobacter* species have fewer repeat protein-coding genes than *E. coli* str. K12 substr. DH108; hence, *Campylobacter* genomes are more compact than that of *E. coli*. In addition, the number of gene families in *Campylobacter* species is smaller than that in *E. coli*; some species even comprise approximately half of *E. coli*, indicating that *E. coli* is more complex than the *Campylobacter* genus. Strains of the *Campylobacter* genus have 27 to 86 genes families with repeat genes (**Table S4**), whereas *E. coli* harbors 242 gene families with repeat genes. The average copy number of repeat genes in the repeat gene families range from 2.16 to 2.80, indicating that the majority of the copies of the repeat genes in the gene families of *Campylobacter* genus are two. Similarly, *E. coli* has 2.36 average copies of repeat genes. The repeat genes may have indispensable and key roles in *Campylobacter* strains. For example, flagellum-related genes are repeated in *C. jejuni* ICDCCJ07001 because flagellum is very important for its mobility, adherence, and pathogenesis. Repeat genes are also utilized for intra-genomic homologous rearrangements [50]. We also determined whether COG is enriched in repeat genes and found that no functional enrichment occurred compared with total genes (p = 0.059). Nevertheless, core genes showed significantly functional enrichment (p = 2.2e-16), indicating enrichment for translation, ribosomal structure, and biogenesis function (COG_J) (**Figure 4**).

Given that evolutionary constraints are multidimensional [51], analysis of a single gene is insufficient to fully understand the phylogenetic relationship of *Campylobacters*. Single gene-based phylogenetic analysis may not reflect their natural relationship because HGT commonly exists in prokaryotes [52], [53]. Thus, we used a genome-wide phylogenetic analysis to definitively determine their relationship. Genome-scale core protein sequence-based method was applied to construct phylogenetic trees for all the 34 collected genomes. Only the orthologous protein sequence with the best hit for homologous genes was used to construct the trees if an organism had multiple copies in one protein family. The conserved regions of all the core proteins were concatenated to construct a phylogenetic tree. A total of 349 core proteins were composed of 84457 amino acid residues (including gaps in some organisms), which accounted for 11.88% (*C. rectus* RM3267) to 20.22% (*C. fetus* subsp. *venerealis* Azul-94) of their total proteins. The neighbor-joining phylogenetic tree based on the conserved region of the core protein sequences (core gene tree) showed similar two lineage clades with the phylogenetic analysis based on SSU rRNA sequences (rRNA tree). However, the intra-lineage relationship of the core gene tree was somewhat slightly inconsistent with that of the rRNA tree (**Figures 2** **and** **5**). Thus, further study is still needed to identify the natural relationship of intra-lineage organisms. Based on the core gene tree, the two strains of *C. coli* were separated from *C. jejuni* organisms. Therefore, the phylogenetic relationship reflected by the core gene tree was more reliable than that reflected by the rRNA tree. The core gene tree can be used as species tree for all of the collected *Campylobacter* species in this study.

**Figure 5:**
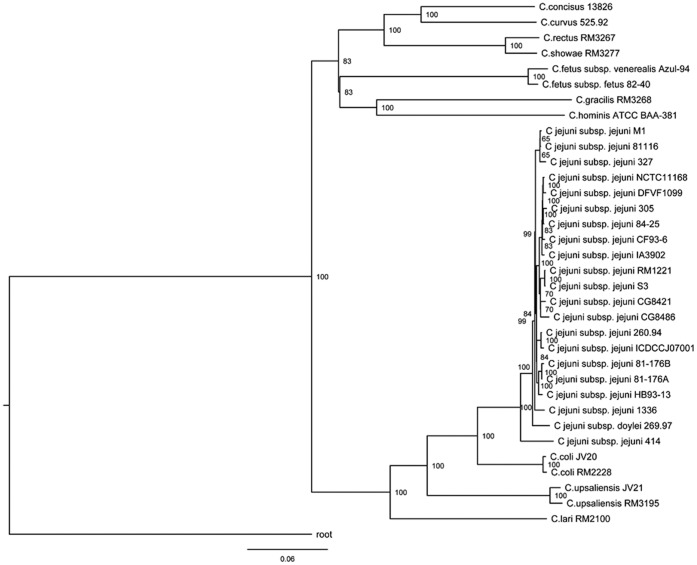
Neighbor-joining phylogenetic analysis of *Campylobacters* based on their core genes. Only the conserved regions of the orthologous core protein sequences were used to construct this genome-wide tree. Bootstrap values (in percent) of 1000 simulations are indicated at all branches. Bar represents 0.06 amino acid substitutions per site.

**Figure 6:**
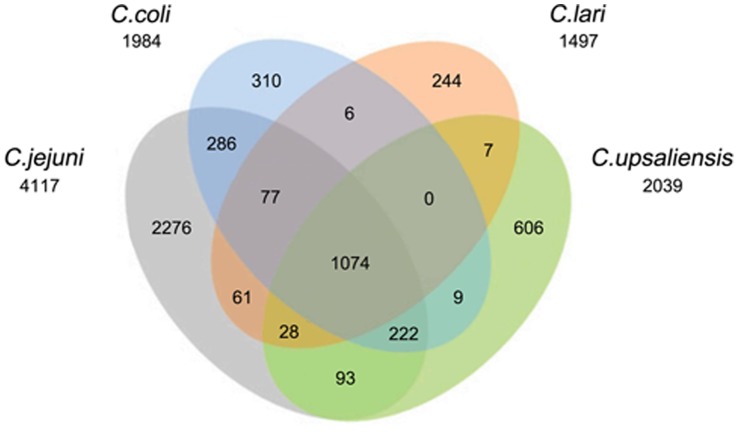
Four-way Venn diagram for gene families of four *Campylobacters*. *C. jejuni*, *C. coli*, *C. lari*, and *C.upsaliensis* from *jejuni* lineage (based on core gene tree) are shown. The absolute numbers of the core and the dispensable and unique gene families of the four species are all shown.

We speculated that a large fraction of gene families may be shared by *C. jejuni*, *C. coli*, *C. lari*, and *C. upsaliensis* because they were constantly embraced in the *jejuni* lineage. As expected, 1074 gene families were shared by these four species, accounting for 26.9%, 54.13%, 71.74%, and 52.67% of their total gene families, respectively (**Figure 6**), indicating that these four species possessed close evolutionary relationship. For *C. jejuni*, the relatively low fraction of gene families entirely shared by these four species was partially due to the large amount of pan-genome resulting from too many strains (up to 21 strains). The fractions of the unique gene families of *C. jejuni*, *C. coli*, *C. lari*, and *C. upsaliensis* were 55.28%, 15.63%, 14.96%, and 29.72% respectively, indicating that *C. jejuni* had a large amount of auxiliary genes. For each species, the majority of the gene families were shared by the other three species (**Figure 6**), confirming the close relationship of these four species. We did not perform a cross-relationship analysis of gene families for the members in *non-jejuni* lineage because of its very diverse lineage.

### Microevolution Determination for *C. jejuni*

**Figure 7:**
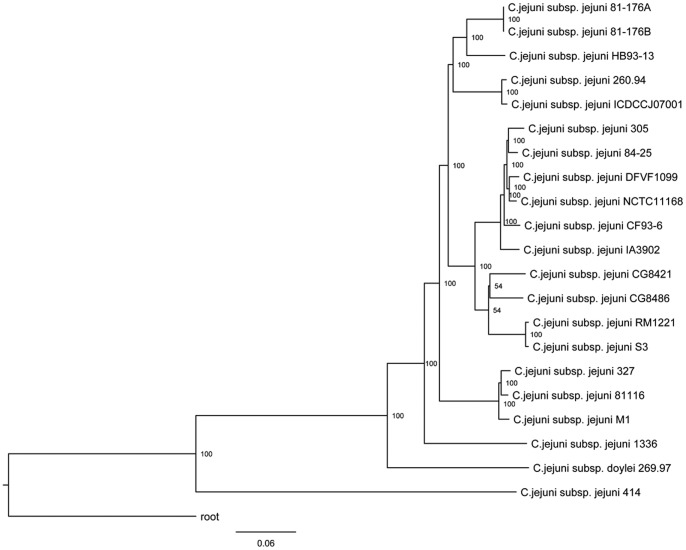
Neighbor-joining phylogeny of *C. jejuni* based on SNP data. The tree is rooted to the outgroup *Campylobacterales bacterium* GD 1. The node support (in percent) after 1000 bootstrap replications is indicated. The scale bar represents 0.06 nucleic acid substitutions per site.

Although the interspecies phylogenetic relationship can be obtained through core gene-based methodology, the precise intraspecies relationship of *C. jejuni* needs further analysis. Both the core gene tree and the rRNA tree had low resolution for the microevolution (intraspecies phylogenetic relationship) of *C. jejuni* because of the high similarity between strains of *C. jejuni* at the genus level. Therefore, SNP information was applied to identify their microevolution relationships. To our knowledge, this study is the first to show the microevolution of *C. jejuni*. A total of 37734 filtered SNPs were obtained, accounting for approximately 0.02% of each genome. Phylogenetic analysis was conducted based on the concatenated SNP sequences. **Figure 7** shows the microevolution relationship of *C. jejuni*. Almost all of the branches had 100% bootstrap support values, indicating that the SNP sequence-based phylogenetic tree was robust and may reflect the natural relationship of *C. jejuni* organisms. As expected, the resolution of the SNP-based tree within the *C. jejuni* was much higher than those of both the core gene tree and rRNA tree. The combination with the core gene tree may determine a comprehensive relationship of all the collected *Campylobacter* genomes. Performing other analyses, such as later gene transfers, is useful when using these two phylogenies as combined control.

### Identification of ACFs

**Figure 8:**
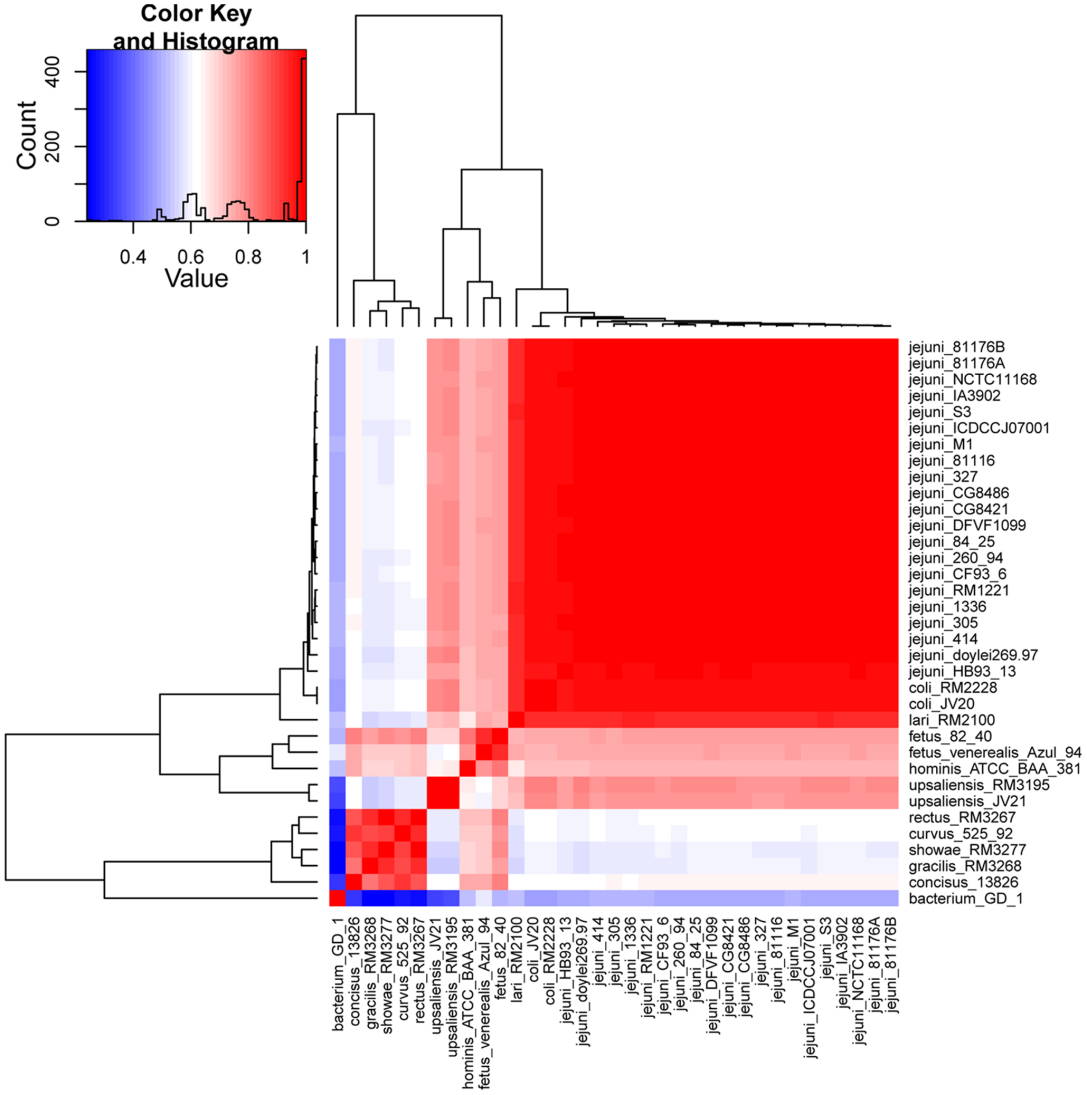
Tetranucleotide usage pattern (TUP) heatmap of the genus *Campylobacter*. The rows and columns stand for strains. Both rows and samples are clustered using ward linkage hierarchical clustering to highlight strain groups with similar genomic signature. The tetranucleotide-derived z-values were predicted using maximum order Markov chain model. Pearson correlations for z-scores were used for clustering and as a basis for color intensity. TUPs of *Campylobacterales bacterium* GD 1 was used as a control.

**Figure 9:**
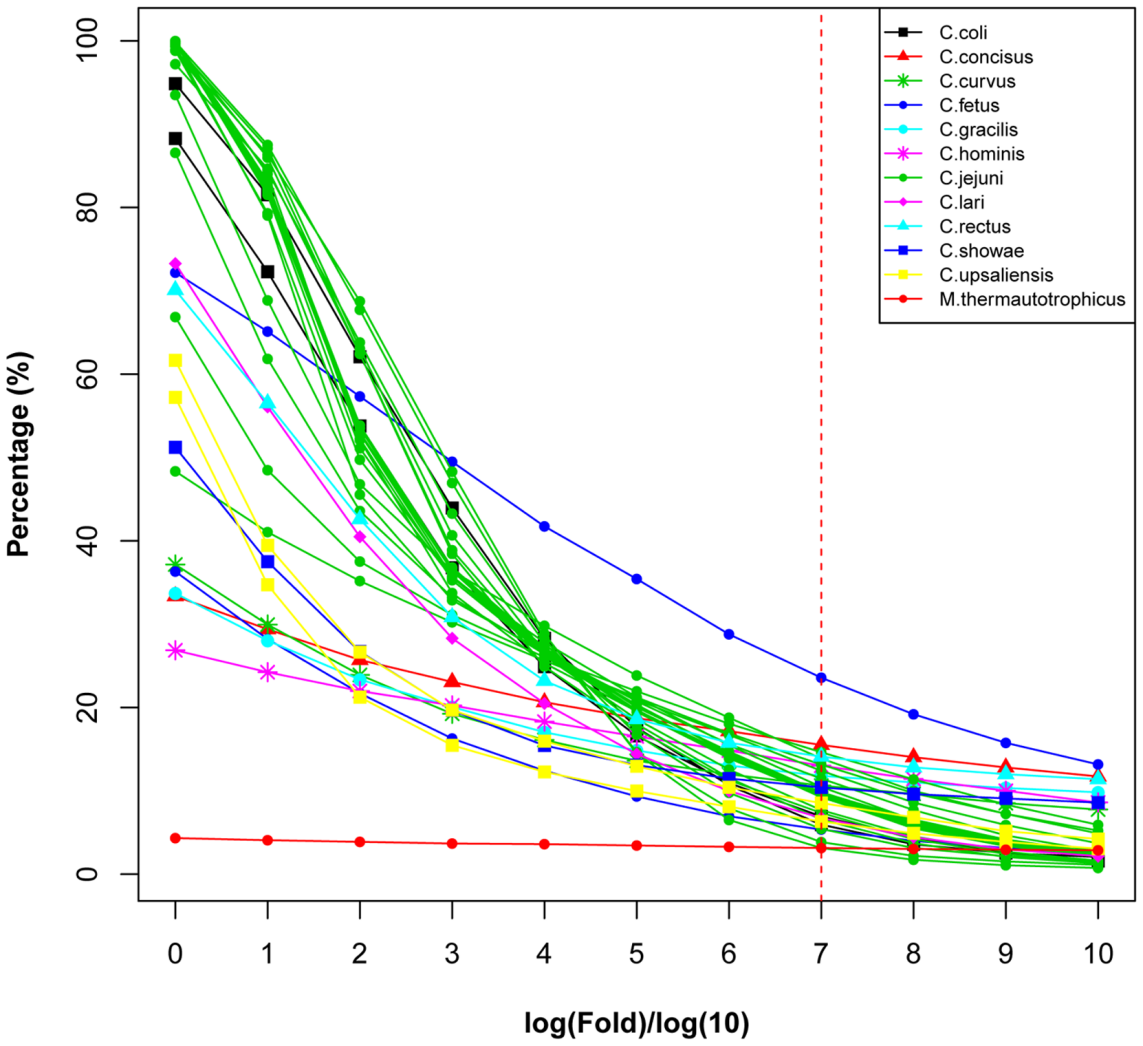
Abnormal composition fragment percentages of *Campylobacter* strains under different folds. *M. thermautotrophicus* str. Delta H (NC_000916.1) was used as control. Fold values (see Methods) were logarithmically transformed. The red dashed line represents the cutoff set in this study for the identified abnormal composition fragments.

*Campylobacters* are genetically diverse according to the aforementioned comprehensive genomic analysis and characterization of all the collected genomes within the genus *Campylobacter*. However, a question on how *Campylobacters* evolved to such diversity is raised. To uncover some clues to this question, we focused on the ACF analysis. The genome-scale TUP of all 34 *Campylobacter* genomes was initially computed, and their relationship was determined (**Figure 8**) to identify the genomic fragments with abnormal composition. An alternative figure was also provided to clearly show their relationship based on the TUP-derived z-values (**Figure S1**, TUP-derived tree) because the genome composition showed somewhat evolutionary implications [54]. The topologies of the TUP-derived tree were significantly different from those of the core gene tree and the rRNA tree (**Figures S1, 2, and 5**). Considering that the core gene tree is an inference tree, the most possible explanation for their incongruities was that all *Campylobacter spp.*were classified into two lineages, similar to that of the core gene tree or rRNA tree. However, *C. fetus* and *C. hominis*, which were included in the *non-jejuni* lineage in the core gene tree or rRNA tree, were transformed from *non-jejuni* lineage to *jejuni* lineage. The reason for this phenomenon is not clearly elucidated and is awaiting further investigation. One possible reason is that TUP evolved more rapidly than SSU rRNA and other core genes in these two species [54]. This hypothesis was partially confirmed based on our results (**Figure 9**), in which the two *C. fetus* genomes possessed significantly different percentages of ACFs, inferring a rapid TUP evolution. In addition, some minor intralineage discrepancies were observed between TUP-derived tree and core gene tree or rRNA tree (**Figures S1, 2, and 5**) because of the different evolutionary rates and later gene transfers.

### Clues for Genomic Diversification

After identifying the global genomic composition features of *Campylobacter* genomes, we subsequently used the modified Naïve Bayesian classifier [46] to search for abnormal genomic fragments. We improved this method using two main aspects: (1) We set a more rigorous cutoff to guarantee that all predicted ACFs under this cutoff were absolutely abnormal enough for HGT prediction; (2) The ACFs were not directly called as HGTs because not all ACFs were HGTs and only certain ACFs that satisfy other additional requirements can be called HGTs. The prediction is very reliable and useful when using the improved Naïve Bayesian classifier.

**Figure 10:**
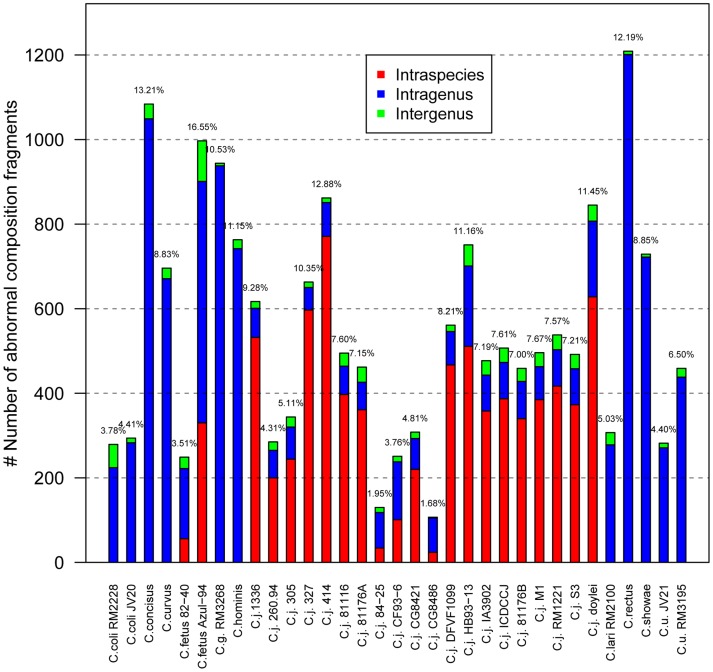
Source of abnormal composition fragments. The values (in percentage) above the bars were calculated as the total abnormal composition fragments divided by the total fragments of the genomes. C.u. = *Campylobacter upsaliensis*; C.j. = *Campylobacter jejuni*; C.g. = *Campylobacter gracilis*; C.j. ICDCCJ = *Campylobacter jejuni* ICDCCJ07001; C.j. *doylei* = *Campylobacter jejuni doylei* 269.97. Strain names of all the other species with only one strain were neglected for simplicity. “intraspecies” stands for abnormal composition fragments coming from other strains of the same species, intragenus means abnormal composition fragments derived from other species of the same genus and intergenus represents abnormal composition fragments originated from other genus.

In a previous study [46], the cutoff for the identification of ACF, called HGTs, was set to 0 (logarithmically transformed). By contrast, we found that almost 100% of the entire genomes for some strains, such as *C. jejuni* subsp. *jejuni* 84–25, had an abnormal composition if the cutoff was set to 0 (**Figure 9**), which seemed impossible. The genome of an archaea *M. thermautotrophicus* str. Delta H [55] was also included in this study to obtain a more reasonable cutoff (**Figure 9**). The line representing *M. thermautotrophicus* str. Delta H was constantly horizontal and intersected the lines of the *Campylobacters* genus at 7 (logarithmically transformed), which can be set for the identification threshold of the ACFs. Previous studies reported that 18% and 24% of the genes may be horizontally transferred in *E. coli* [56] and *Thermotoga maritime* [57], respectively. Under the cutoff of 7, a collection of fragments (500 bp) was identified as ACFs, accounting for 1.15% to 13.21% of their genomes (**Figure 10**), comparable to previous studies [57], [58]. These results suggested that our selective threshold was reasonable and rigorous, and the resultant ACFs were reliable. In addition, the most possible origins of these fragments were also analyzed. As shown in **Figure 10**, some species, for example *C. rectus*, which only had one strain in this study, had no intraspecies origin. *C. jejuni* harbored high fraction of intraspecies-derived ACFs, whereas other *Campylobacter* strains had high fraction of intragenus-derived ACFs, which may be imported from other species at the same genus other than the same species. Therefore, high intraspecies recombination or HGT events may mainly occur in *C. jejuni*, whereas high intragenus recombination or HGT events may mainly occur in other *Campylobacter* strains. To some extent, this phenomenon can explain why *Campylobacters* were genetically and phenotypically diverse.

*De novo* re-prediction genes including rRNA, tRNA, and protein-coding genes were formerly carried out. Consecutive fragments, which possessed at least two fragments with fold larger than 7 (logarithmically transformed), were concatenated into genomic regions (GRs). The gene types of GRs were subsequently analyzed (summarized in **Table S4**). Most of the rRNA- and tRNA-coding genes considered as housekeeping genes had abnormal compositions, indicating that the abnormal composition genes were horizontally transferred or acted as housekeeping genes through vertical inheritance during the evolutionary process. Therefore, we preferred the ACFs than the horizontally transferred genes in this study [46]. The results also showed that some protein-coding genes were also housekeeping genes rather than horizontally transferred genes (**Table S3**). A total of 3783 gene families had abnormal composition genes. In this study, the gene families residing equal to or larger than 90% of *Campylobacter* genomes were defined as housekeeping gene families. The genes from the housekeeping gene families were defined as *Campylobacter* housekeeping genes. A total of 3738 protein-coding gene families were considered to have abnormal fragments according to our modified method. Among these families, 669 were housekeeping gene families, accounting for 17.68% (669/3783) of the total gene families with abnormal composition genes. Other 82.32% (3114/3783) of gene families may be horizontally transferred genes (**Table S5)**. **Table S4** shows the percentage and absolute number of the housekeeping protein-coding genes and the horizontally transferred protein-coding genes. Housekeeping genes were not horizontally transferred genes, but possibly transferred to the common ancestor of all *Campylobacters*. Therefore, some housekeeping genes may be transferred a long time ago, at least before *Campylobacter* species differentiation.

*Campylobacter* genomes were reported to harbor phage-like genomic fragments [30] or undergo recombination under bacteriophage predation [59]. Therefore, virus or phage may have an important role in *Campylobacter* genome diversification. Viral and phage homoplasies of *Campylobacters* were thus identified. We initially thought that almost all virus-related and phage-related protein-coding genes should be included in the abnormal composition gene list because these homoplasies were foreign. Unexpectedly, for almost all the genomes, the vast majority of the virus-related and phage-related protein-coding genes were not included in the abnormal composition gene list; some genomes had no virus- or phage-related genes with abnormal composition (**Table S6**). Intergenomic differences were generally higher than intragenomic differences [60], and nearly all the native GRs within a certain genome had similar but organism-specific genomic composition. Consequently, GRs recently introduced from other organisms showed unusual sequence characteristics and can be distinguished from the recipient genome. However, imported GRs will be ameliorated to reflect the genomic characteristics of the recipient genome other than the donor genome after a long period of mutation because the introgressed genes were subjected to the same mutational processes affecting all genes in the recipient genome [61]. Viral or phage genes with similar genomic composition to their genomes were suggested to have transferred into the host genomes of genus *Campylobacter* a long time ago, indicating that an ancient viral integration occurred in the *Campylobacter* genomes. However, other genomes still recently possess abilities to acquire viral or phage genomic fragments (**Table S6**). For example, in *C. concisus* 13826, the majority of the phage-related genes may have been recently transferred into its genome because 76.47% of the phage-related genes were included in the abnormal composition gene list. This result implied that some genomes still have viral or phage integration abilities. This phenomenon explains, to some extent, why *Campylobacter* genomes are diverse. Therefore, we concluded that some *Campylobacter* genomes had undergone certain ancient vital integration during their evolution.

## Supporting Information

Figure S1 **Clustering result based on tetranucleotide usage patterns of the genus** ***Campylobacter*** **.** Bar represents the clustering height. *Campylobacterales bacterium* GD 1 (termed *Campylobacterales* in picture) was used as the outgroup. Pearson correlations for tetranucleotide-derived z-values were used for clustering the genomes using ward linkage hierarchical algorithm.
(TIF)

Table S1 ***Campylobacter*** **genomes examined.** Thirty-four *Campylobacter* genomes collected for analysis were listed with their genomic information including GenBank GI number, mark for genome in this study, contig number, total length, and SSU rRNA number.
(XLSX)

Table S2 **Genome-wide alignment matrix of genus** ***Campylobacter*** **.** Genome-wide alignment similarity ratio in the table cells was computed as the length of genome fragments preserved between any two organisms against the length of total genomes of the organism in the row.
(XLSX)

Table S3 **Gene classification with abnormal composition.** Genes with abnormal composition were classified into groups of different gene types including rRNA, tRNA, and protein-coding genes (coding sequences, CDS). The CDS was further divided into housekeeping CDS and HGT CDS. The gene number and the percentage to total gene number were listed for these genes.
(XLSX)

Table S4 **Gene families containing duplicated genes in genus** ***Campylobacter*** **.** For each *Campylobacter* organism, the number of gene families containing duplicated genes and the total number of duplicated genes, together with average gene copies, (average gene number of all its gene families containing repeat genes) are shown.
(XLSX)

Table S5 **Gene family information of abnormal composition genes.** The numbers in table cells represent the number of homologous genes in the corresponding genome. The Gene ID of the first column had the longest sequence in the gene family.
(XLSX)

Table S6 **Quantitative composition of** ***Campylobacter*** **virus-related and phage-related genes containing abnormal composition fragments.** The percentage is equal to the number of virus- or phage-related genes with abnormal composition divided by the number of the total virus- or phage-related genes.
(XLSX)
