# Supplementary figures and images for: Comprehensive Genomic Characterization of Campylobacter Genus Reveals Some Underlying Mechanisms for its Genomic Diversification
Source: PLoS One. 2013 Aug 5;8(8):e70241. doi: 10.1371/journal.pone.0070241 (PMC3734277; doi:10.1371/journal.pone.0070241)

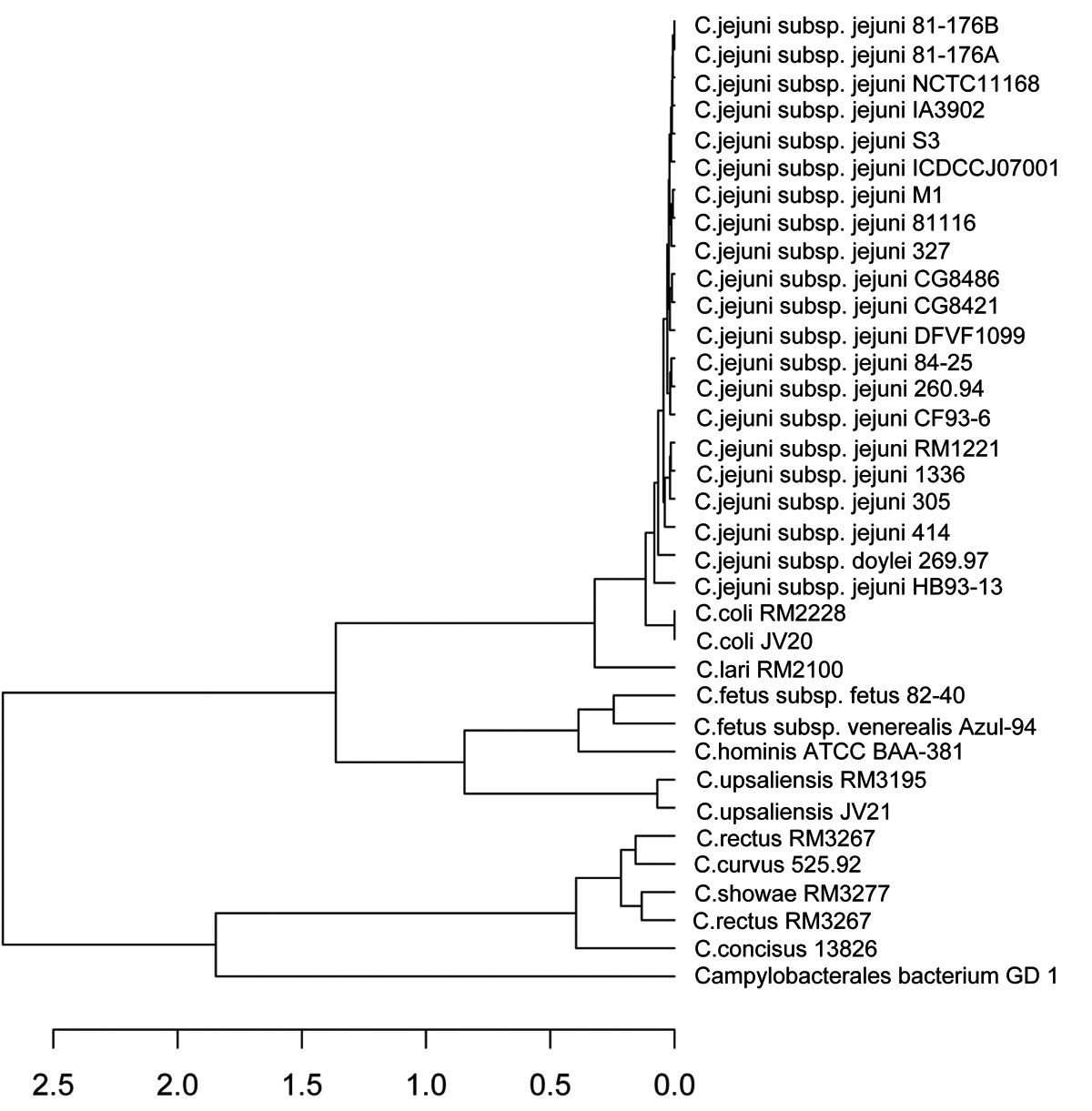

Supplement: Figure S1 — Clustering result based on tetranucleotide usage patterns of the genus Campylobacter . Bar represents the clustering height. Campylobacterales bacterium GD 1 (termed Campylobacterales in picture) was used as the outgroup. Pearson correlations for tetranucleotide-derived z-values were used for clustering the genomes using ward linkage hierarchical algorithm. (TIF) [file pone.0070241.s001.tif]
